# Hyperglycemia-induced oxidative stress and epigenetic regulation of ET-1 gene in endothelial cells

**DOI:** 10.3389/fgene.2023.1167773

**Published:** 2023-04-17

**Authors:** Dalal Nasser Binjawhar, Alaa T. Alhazmi, Wejdan Nasser Bin Jawhar, Walaa MohammedSaeed, Sher Zaman Safi

**Affiliations:** ^1^ Department of Chemistry, College of Science, Princess Nourah Bint Abdulrahman University, Riyadh, Saudi Arabia; ^2^ Hera General Hospital, Makkah, Saudi Arabia; ^3^ Faculty—Clinical Laboratory Sciences Department, College of Applied Medical Sciences, King Saud University, Riyadh, Saudi Arabia; ^4^ Department of Medical Laboratory Technology, Faculty of Applied Medical Science at Taibah University, Al Madinah Al Munawarah, Saudi Arabia; ^5^ Faculty of Medicine, Bioscience and Nursing, MAHSA University, Selangor, Malaysia

**Keywords:** ET-1, gene expression, methylation, hyperglycemia, oxidative stress

## Abstract

**Introduction:** Hyperglycemia-induced endothelial dysfunction and the subsequent increase of oxidative stress could lead to aberrant regulation of various genes which are responsible for a range of functions. This study aims to find out how hyperglycemia affect oxidative stress and then the expression and methylation of endothelin 1 (ET-1) gene in in human umbilical vein endothelial cells (HUVEC).

**Methods:** Cells were cultured in growth medium and exposed to low and high glucose concentrations to mimic normal and diabetic condition respectively. Computational analysis were performed using UCSC genome browser and eukaryotic promoter database (EPD). The expression of ET-1 gene was investigated by real time PCR. Cytotoxicity and oxidative stress were determined by MTT and DCFH-DA assays respectively. Promoter methylation was assessed by the bisulfite sequencing method.

**Results:** DCFH-DA assay showed that hyperglycemia can significantly increase the regulation of reactive oxygen species synthesis. The relative expression of ET-1 gene was increased due to exposure to high glucose concentration. MTT assay revealed reduced viability of cells due to the glucose induced damage. Methylation analysis revealed hypomethylation of the promoter of ET-1 however the difference was not significant. Out of 175 CpGs at 25 CpG sites, only 36 CpGs were methylated (20.5% methylation) in cell treated with normal glucose. Upon exposure to high glucose only 30 CpGs were methylated in 175 CpGs at 25 CpG sites (17.1% methylation).

**Discussion:** Our study concludes a significantly high expression of ET-1 gene in response to high glucose exposure in HUVECs. It also reports that hyperglycemic condition leads to elevated oxidative stress. No significant change was found in methylation when cells were treated with high and low glucose concentrations.

## Introduction

Diabetes mellitus (DM) is a multifactorial disease in which body fails to release or respond to insulin. The increasing incidence of diabetes is a growing concern, causing a huge death toll and disability worldwide (Safi et al., 2014a; Barysheva, 2015; Shestakova and Maioriv, 2019; Darenskaya et al., 2021). In diabetes, chronic hyperglycemia consequently contributes to the progression of several other diabetic complications including endothelial dysfunction, insulin resistance, nephropathy and retinopathy (Safi et al., 2014b; Cho et al., 2018). Vascular endothelium performs a range of functions including maintenance of vascular homeostasis and regulation of adhesion molecules. The condition in which endothelium fails to maintain vascular homeostasis is known as endothelial dysfunction (Sena et al., 2013; Cai et al., 2021).

Under hyperglycemic conditions, the normal vascular physiology changes to endothelial dysfunction, which leads to dysfunctional cellular redox and increased generation of oxidative stress (Meza et al., 2019). Increased ROS generation leads to aberrant regulation of a range of genes including inflammatory cytokines and adhesion molecules. In response to hyperglycemic environment, several pro-inflammatory pathways also participate during the process of oxidation and antioxidation. Consequently, the imbalance of enzymatic and non-enzymatic antioxidant and ROS generation induce endothelial dysfunction by augmenting endothelium permeability, necrosis and apoptosis in endothelial cells (Batumalaie et al., 2013; SZ Safi et al., 2015; Safi et al., 2016; Escobar-Morreale et al., 2017; Luna and Estévez, 2018; Safi et al., 2022; Wang et al., 2022).

Endothelin 1 (ET-1) gene is a very important vasoconstrictor gene which is primarily expressed in vascular endothelial cells. Due to its role as vasoconstrictor, this gene contributes to vascular remodeling in diabetes and other diabetic complications. ET-1 is reported to play a key role in endothelial dysfunction (du Plooy et al., 2017; Kruger et al., 2012; Akter et al., 2015). ET-1, TNF-α and angiotensin II are some of the inducing factors of superoxide radical (O2−) generation (Münzel et al., 2010; Montezano and Touyz, 2012). In endothelial dysfunction, various studies have demonstrated the role of ET-1 gene in augmenting ROS and increased production of O_2_- in human arteries and animal vessels (Elmarakby et al., 2005; Loomis et al., 2005; Böhm et al., 2007; Cerrato et al., 2012; Sánchez et al., 2014). Studies have also shown that blocking the ET receptor improves endothelial function in human coronary arteries (Verma et al., 2001; Romero et al., 2009).

Epigenetics is an emerging field in which epigenetic modification can regulate genes without changing their sequences. These are heritable and stable modifications which alter the gene function without changing the DNA sequence (Pradhan et al., 2016; Álvarez-Nava and Lanes, 2018; Ling and Rönn, 2019). In diabetic complications, a number of studies have found alterations in DNA methylation which could change the gene expression profiles, and consequently the fate of the disease (Rakyan et al., 2011; Volkmar et al., 2012). One study reported hypomethylation in DNA from liver tissue (Williams et al., 2008). Another study on diabetic rat model found increased DNA methylation in pancreatic tissue. This study concluded that methylation and demethylation of DNA in diabetes may attribute to various factors the local conditions and type of tissue exposed to the disease (Williams and Schalinske, 2012).

Keeping in view the above reports, and the role of epigenetics, the purpose of this study was to assess the level of methylation and its association with the expression of ET-1 gene. We also aimed to investigate the association of hyperglycemia and the level of reactive oxygen species in hyperglycemic human umbilical vein endothelial cells (HUVEC). To this end, we exposed the cells to high and low glucose concentrations and evaluated the expression of ET-1 gene. We also studies the methylation level in ET-1 gene to establish its role in hyperglycemic condition and probe the possibility of any association between gene expression and methylation in cells with high oxidative stress.

## Methodology

### Cell culture

Using 10% serum and 1% Penicillin/Streptomycin, human umbilical vein endothelial cells (HUVEC) were cultured in a complete growth medium (Cell Systems). Cells were grown in 25 and 75 ml flask. One set of cells was treated with normal glucose (5 mM, 0.9008 g/L) while the other was exposed to high glucose concentration (25 mM, 4.5 g/L). Upon 80% confluency, cells were passaged using the same formulation of growth medium. Cells were incubated for 24 and 48 hours in 5% CO_2_ at 37°C and media was changed every 2–3 days. Cells viability and growth was observed everyday using microscope. Cells were sub-cultured by detaching the cells by Trypsin-EDTA (Gibco® United States). RNA and DNA were extracted using commercially available kits (Gene JET and Qiagen). The cell lines present in this study were obtained from Thermo Fisher Scientific, United States (C0155C).

### MTT assay

MTT assay was conducted following an already reported method (Kumar et al., 2018) with modifications. Briefly, 96 well plates were used to seed the cells containing experimental (24 and 48 h) and control (24 and 48 h) groups, treated with high and low glucose concentrations. In each well, a total of 1 × 10^4^ human umbilical vein endothelial cells were cultured for 24 and 48 h followed by incubation at 37°C with 5% CO_2_. Experiments were initiated with addition of MTT reagents in triplicates, followed by incubation for 4 h. Cells were rendered to extensive wash, and solubilization solution was added to dissolve the formazan crystals. A BIO-RAD-PR 4100 microplate reader was used to take readings at 570 nm after 24 and 48 h incubation.

### Real time PCR

RNA was isolated from high and low glucose treated cell lysates using commercially available Gene JET RNA Purification kit (Thermo Scientific, United States). RevertAid™ kit (Thermo Scientific, United States) was used to synthesize cDNA. ET-1 gene was amplified by real time PCR using Maxima SYBR Green PCR master mix from Thermo Scientific, United States. Ct and ΔΔCt method along with beta-actin as control, was employed to calculate the relative expression of ET-1 gene.

### DCFH-DA assay

To investigate the level of reactive oxygen species (ROS), reagent 2′7′-dichloro-dihydro-fluorescin diacetate was added to the experimental and control cells with high and low glucose concentrations, respectively. Cells were seeded in 96-well plates and 5 μM DCFH-DA reagent was added, followed by incubation for 24 and 48 h. Fluorescence plate reader was used to take readings at 530 nm emission and 485 nm excitation. For the calculation of reactive oxygen species, the mean control was deducted from the mean experimental group.

### Computational analysis–Primer design and selection of sequences

**FIGURE 1:**
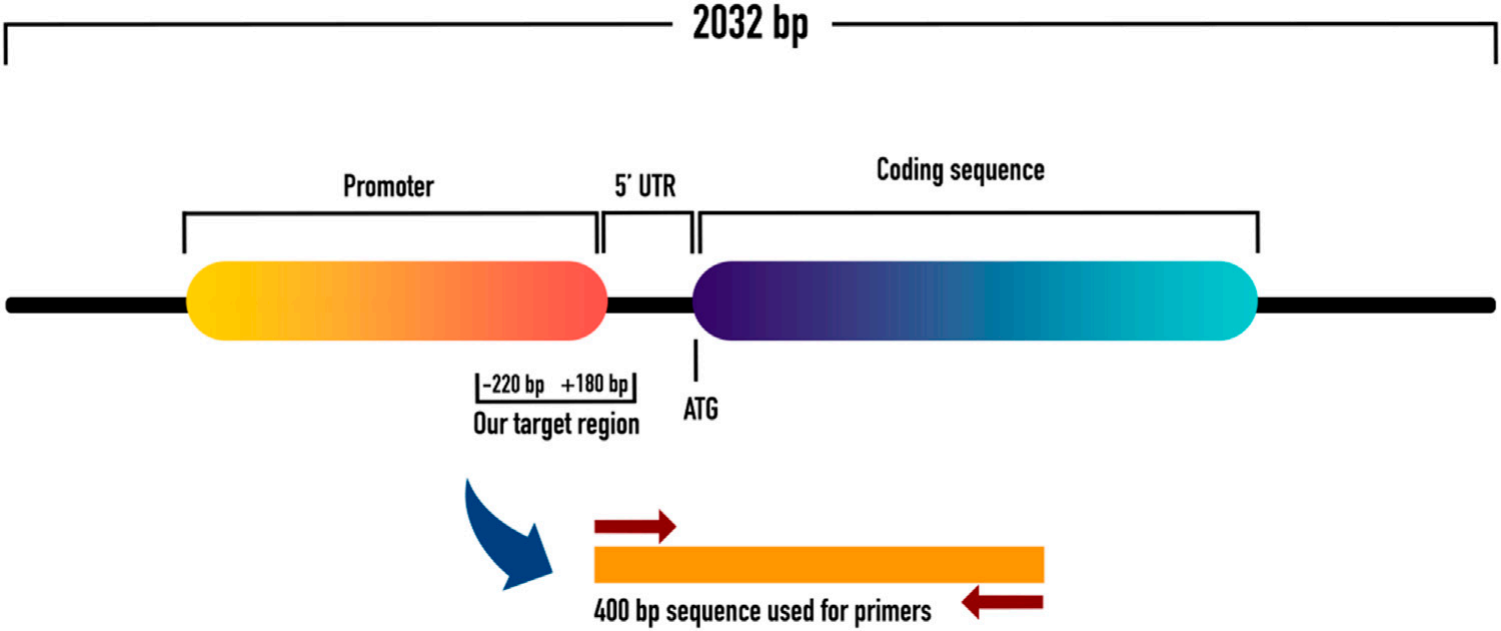
Shows the schematic representation of ET-1 with coding and promoter sequences. It also shows the 5′UTR with downstream and upstream regions from where we picked our sequence of interest for methylation analysis.

**FIGURE 2:**
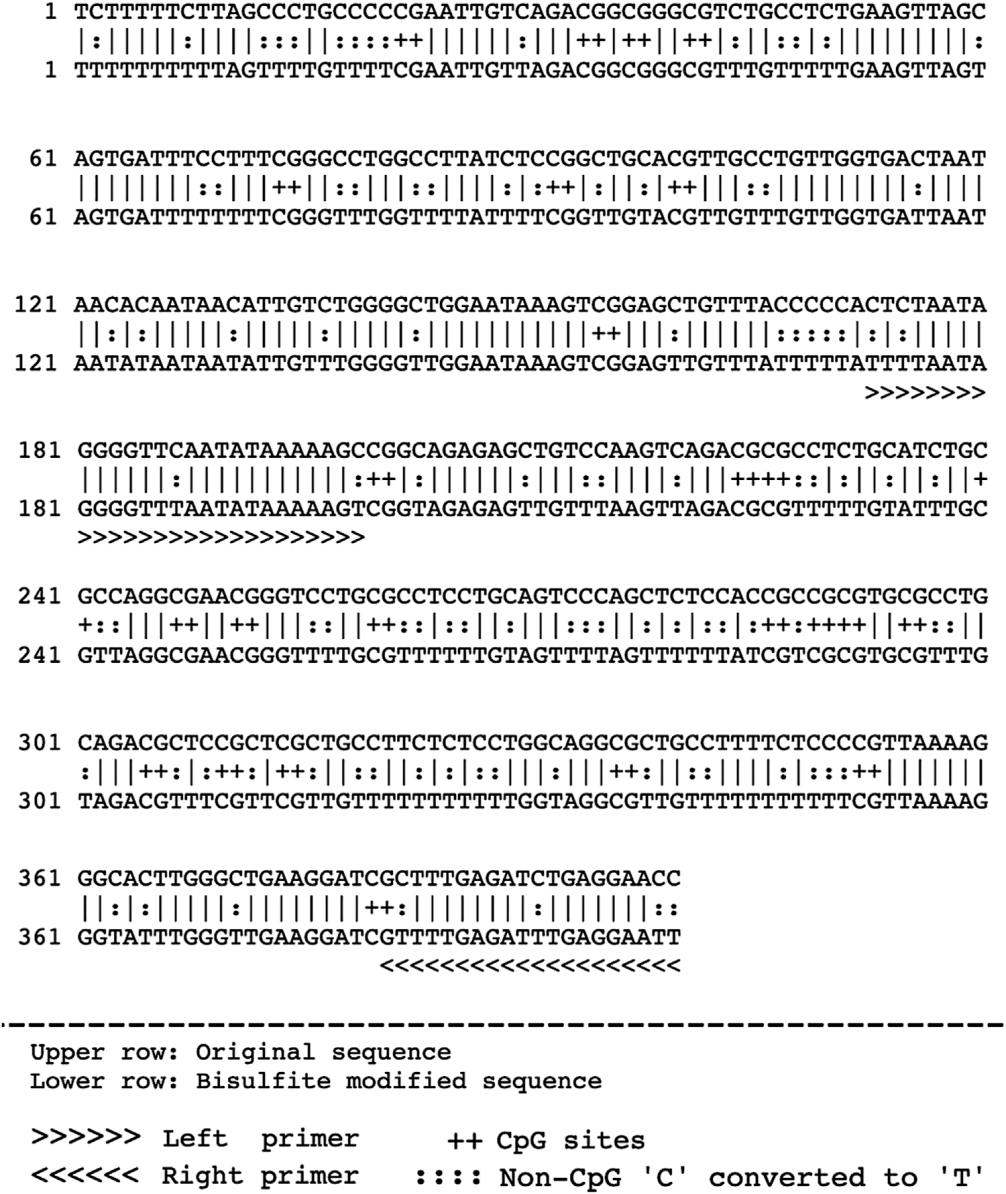
Shows the 400 bp sequence which contained a 180 pb downstream sequence (relative to TSS) within the 5′ UTR, and −220 bp upstream sequence (relative to TSS). This 400 bp sequence was used to design methylation primers for methylation study.

To evaluate the promoter methylation of ET-1 gene, the gene transcript which consisted of 2032 bp, was retrieved from NCBI and UCSC genome browser databases. The 2,032 bp transcript of ET-1 gene had a 636 bp coding region, in which 269 bp was 5′UTR and 1,127 bp 3′UTR. Relative to transcription start site (TSS), promoter sequences (from 180 downstream to −1000 upstream) were identified using Eukaryotic Promoter Database (EPD). Out of 1180 bp long promoter sequence (from 180 downstream to −1000 upstream) a 400 bp sequence, with appropriate number of CpGs was identified for primer designing. The 400 bp sequence contained a 180 pb downstream sequence, relative to TSS within the 5′ UTR. The remaining 220 bp sequence was from the upstream region (−220) relative to TSS. Subsequently, primers were designed within the 400 bp sequence using MethPrimer tool, as shown in Figures 1, 2.

### PCR amplification and bisulfite conversion 

After quantification, a total of 500 ng of extracted DNA was bisulfite-converted using commercially available EZ DNA kit (Zymo United States). Bisulfite modified DNA was amplified using DNA polymerase (hot start taq) with specially designed primers for the promoter of ET-1 gene. PCR reactions conditions were set at 95°C for 5 minutes followed by 95°C for 45 sec and 60°C for 60°s. The final extension was given for 7 minutes at 72°C. Electrophoresis was carried out using agarose gel. A commercially available mini elute gel extraction kit (Qiagen) was used to purify the DNA from the agarose gel.

### Cloning, transformation and sequencing 

After purifying the amplified product from gel, ligation into pCR2.1 vector was initiated using a cloning kit from Invitrogen. A 4 μl of PCR product was thoroughly mixed with 1 μl of TOPO vector followed by a 5 minutes incubation at room temperature. The reaction was again incubated on ice for 10 minutes after adding chemically competent DH5α cells. It was followed by heat shock procedure for 30 s. To check the cloning, all samples were spread onto LB plates and incubated overnight at 37°C. After blue-white screening, 10 colonies of both types of samples (high and low glucose concentration for 48 h) were isolated from the LB plates. The isolated colonies were cultured using ImMedia Amp Liquid for 24 h. Plasmids were isolated using commercially available kit (Favorgen). The final samples were stored for sequencing. For methylation studies, we included only 48 h exposure (the maximum) and 24 h exposure was not included.

### Methylation analysis

Out of 10 samples for each, we encountered cloning/sequencing problems in 3 samples. After deducting 3 samples, we were left with 7 samples for each control and high glucose treated sample. Different softwares and platforms including QUMA (Quantification tool for methylation analysis), NCBI and UCSC genome browser were used to analyze the data.

### Statistical analysis

GraphPad prism and SPSS were used to for the statistical analysis. The results were calculated in mean SD. Statistical significance was determined using multiple *t* tests. Significance was set at a value of 0.05.

## Results

### Effect of high glucose on the proliferation and viability of cells

**FIGURE 3:**
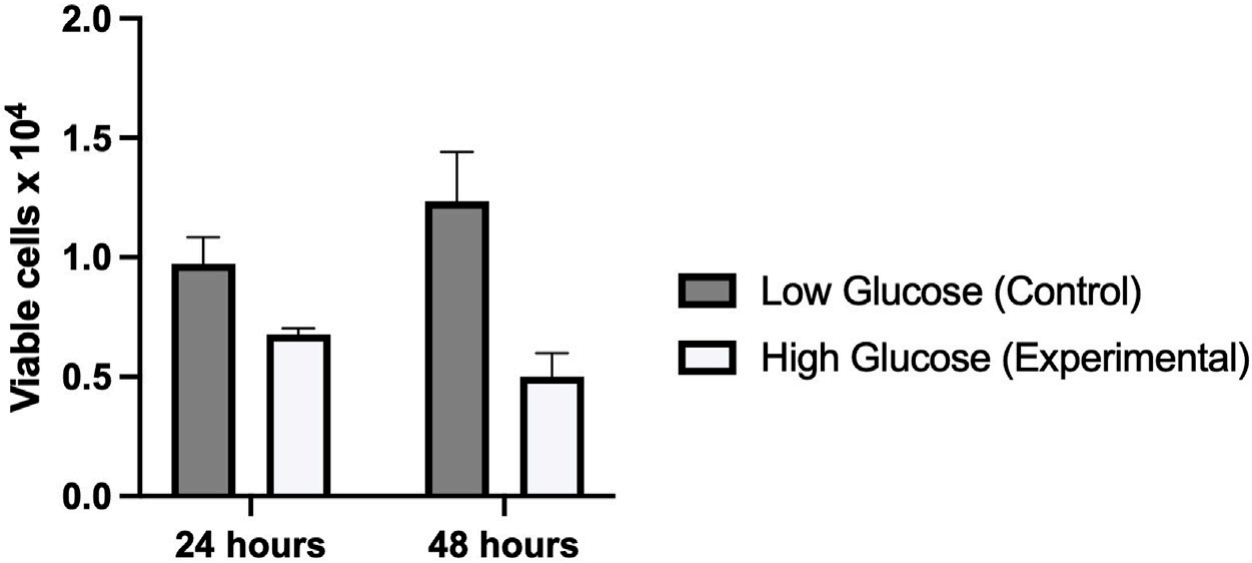
Graphical representation of MTT assay which shows a significantly reduced viability of cells treated with high glucose as compared to low glucose for 24 and 48 h. The viability further reduced in high glucose treated for 48 h.

To see the effect of high glucose on HUVEC, MTT assay was performed in cells exposed to physiological glucose (5 mM), and High glucose (25 mM) concentrations. As shown in Figure 3, MTT assay revealed a significantly decreased cell proliferation index in cells treated with high glucose (25 mM) as compared to control (5 mM). Cells treated for 48 h showed far less viability (*p*-value 0.005) than cells treated for 24 h (*p*-value 0.010) It suggests that hyperglycemia creates a deleterious condition in the cells which doesn’t favor a healthy cell proliferation.

### Expression of ET-1 in hyperglycemic HUVECs

**FIGURE 4:**
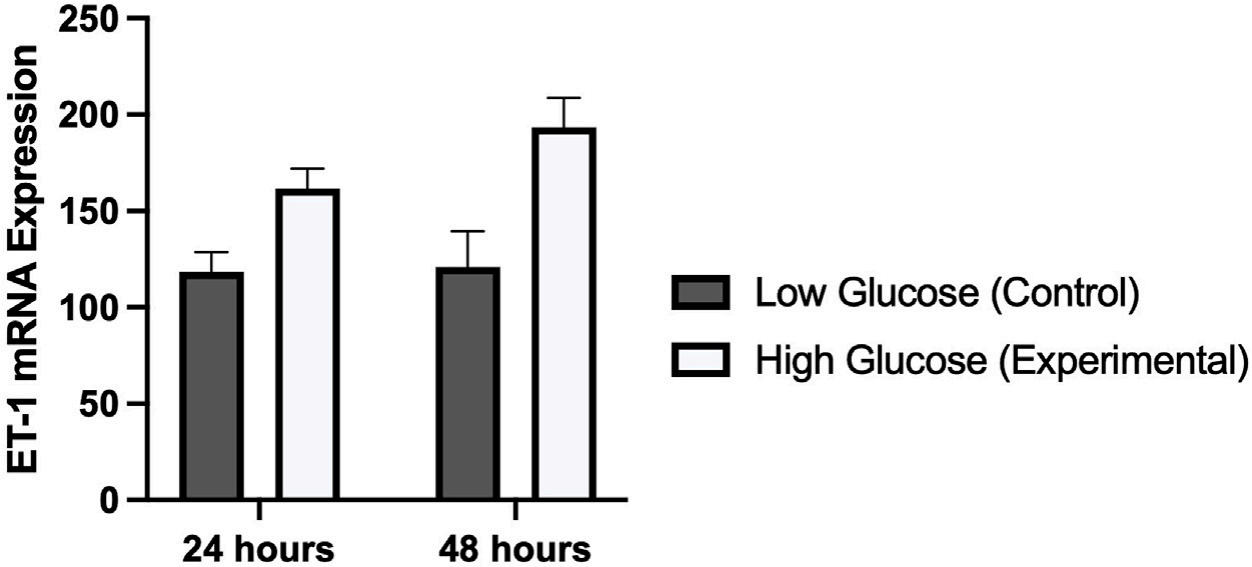
Graphical representation of the mRNA expression of ET-1 gene using real time PCR in HUVECs treated with normal/low glucose and high glucose concentrations for 24 and 48 h. There was a time dependent increased in the expression of ET-1.

Results from real time PCR showed a significantly higher expression of ET-1 gene in HUVECs treated with high glucose concentration as compared to normal physiological concentration (Figure 4). Difference in expression was slightly higher in cells treated for 48 h (*p*-value 0.006) as compared to cells treated for 24 h (*p*-value 0.007). These results of cell viability from MTT assay, and ET-1 expression from real time PCR exhibited a similar time dependent pattern.

### Reactive oxygen species (ROS)

**FIGURE 5:**
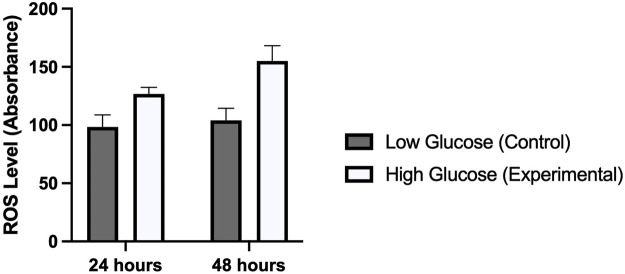
Shows the level of reactive oxygen species in HUVECs treated with normal/low glucose and high glucose concentrations for 24 and 48 h.

Several studies have reported a strong correlation between hyperglycemia and elevated levels of reactive oxygen species. We were interested to see this in human umbilical vein endothelial cells which were treated with high and low concentrations of glucose for different time periods (24 and 48 h). Our results demonstrated a significantly high oxidative stress in cells treated with high glucose as compared to those which were treated with normal glucose concentration. ROS levels were comparatively high in those cells which were exposed for 48 h (*p*-value 0.006) as compared to 24 h (*p*-value 0.014). This was consistent with the expression of ET-1 and cell viability in MTT assay (Figure 5).

### ET- 1 gene methylation and associated factors

**FIGURE 6:**
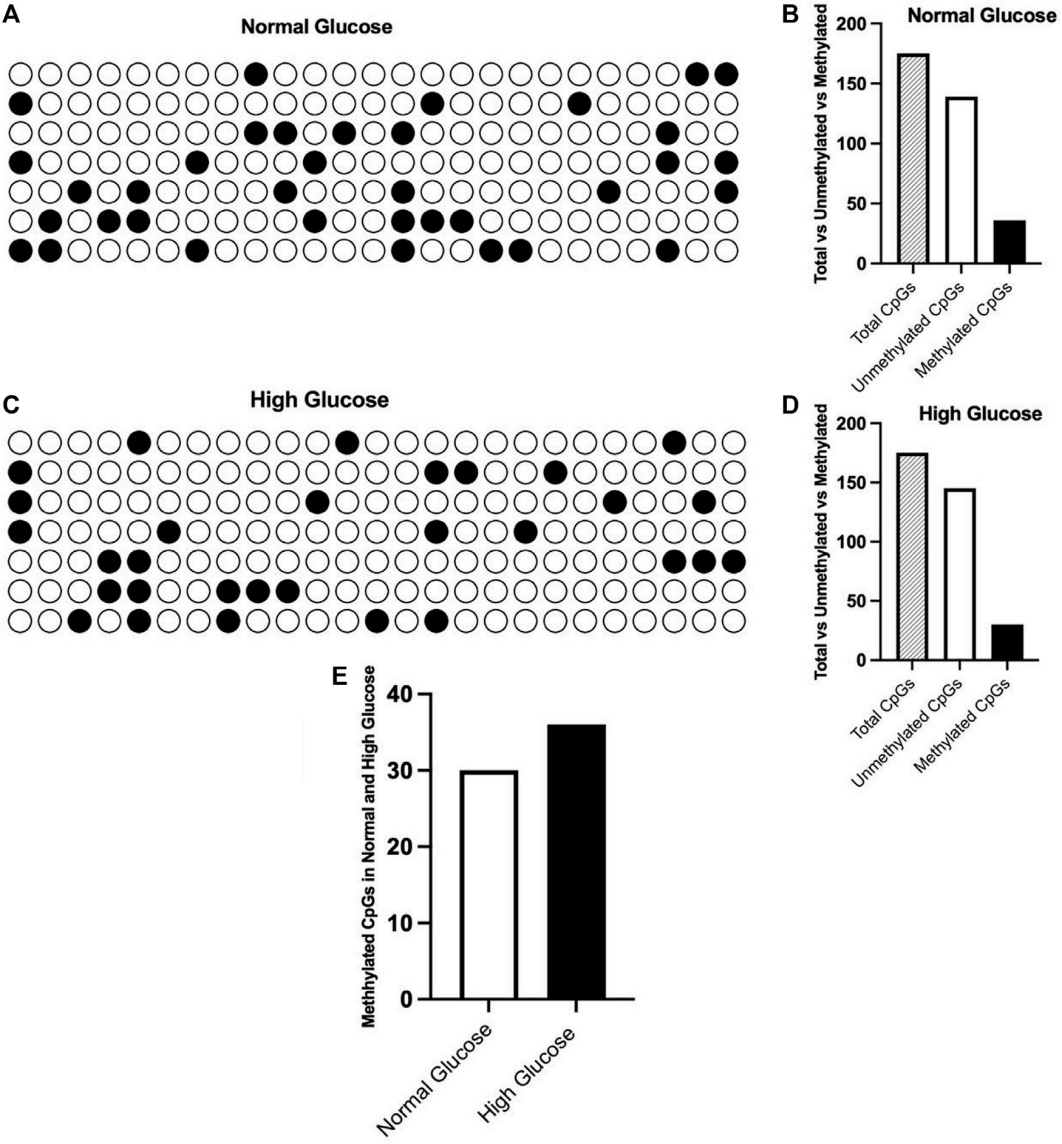
Shows the overall promoter methylation of ET-1 gene. **(A)** methylation of ET-1 gene at normal glucose concentration. **(B)** it summarizes the methylated vs. unmethylated CpGs in the promoter of ET-1. **(C)** methylation of ET-1 gene at high glucose concentration. **(D)** methylation vs. unmethylated CpGs in high cells treated with high glucose concentration. **(E)** the difference of methylation in HUVECs treated with normal and high glucose concentrations.

After we detected a significantly high ET-1 expression in HUVECs, we presumed a hypomethylation in the promoter of ET-1. To check our hypothesis we carried out promoter methylation analysis. We evaluated 25 CpGs in each sample, where we had 7 samples for each high glucose and low glucose treated cells. So we investigated a total of 175 CpGs in high glucose treated cells and 175 CpGs in low glucose treated cells (control). As shown in Figures 6A, B, we found 36 methylated CpGs out of 175 CpGs in HUVECs treated with normal glucose concentration. In high glucose treated cells, the number of methylated CpGs was 30 (Figures 6C, D). Contrary to our expectations, the hypomethylation in glucose treated cells was very trivial and insignificant (Figure 6E). This demonstrates that hyperglycemia and high oxidative stress may regulate the expression of ET-1 gene without involving methylation at its promoter sequence. Cells with low glucose concentrations exhibited high methylation at CpG positions 22, 251, 236 and 380. At positions 92, 155, 247, 295 and 314, no methylation was found. Cells treated with high glucose concentrations, showed high methylation at positions 22, 74 and 260. No methylation was detected at positions 34, 100, 251, 289 and 305.

## Discussion

Hyperglycemia and reactive oxygen species are the hallmarks of diabetes and diabetic complications. In this study we demonstrate that a 24 and 48 h exposure of HUVECs to high glucose significantly alter the ET-1 gene expression. It also negatively affect the viability of cells with increased level of reactive oxygen species. Hyperglycemia caused a significant reduction in cell viability which suggested that glucose has created a pro-oxidative stress environment. In such diabetic and high oxidative stress conditions, studies have shown that pro-diabetic, pro inflammatory and pro-oxidative stress genes are highly expressed. Several studies have reported altered gene expressions in such conditions (Shaik et al., 2010; Safi et al., 2018; Bima et al., 2022a; Aziz et al., 2022; Bima et al., 2022b). In a recent study, Hall et al. reported a significant impact of high glucose exposure on global gene expression and DNA methylation in human pancreatic islets (Hall et al., 2018). Several genes including VAC14, RASD1, SYT16, CHRNA5 and TMED3 showed altered gene expression in response to high glucose exposure. Another study reported distinct patterns of gene expressions in primary human skin cells treated with normal and high glucose concentrations (Zhang et al., 2021).

A number of studies have reported altered methylation patterns in response to high glucose exposure. In 2020, Kandilya et al. reported varied levels of DNA methylation in human neural progenitor cells (Kandilya et al., 2020). Several other studies have also concluded that altered DNA methylation and other epigenetic mechanisms play an important role in the pathogenesis of diabetes and other diabetic complications (Ling et al., 2008; Yang et al., 2011; Wu et al., 2016; Ahmed et al., 2020; Chen et al., 2021). Similarly, elevated levels of both reactive oxygen species (ROS) and DNA methylation have been reported to be the characteristics of various diseases including cancer, diabetes, endothelial dysfunction and atherosclerosis (Wu et al., 2016). Several studies have reported a possible association of elevated oxidative stress and epigenetic modifications (Galligan et al., 2014; Wu and Ni, 2015; Khan et al., 2016; Kreuz and Fischle, 2016; Zhou et al., 2016; Zhou et al., 2018).

In diabetic patients, high blood glucose and elevated oxidative stress contribute to affect the epigenetic landscape, thus leading to persistent upregulation/downregulation of genes controlling vascular homeostasis. A significant shift in gene expression profiles also point to an altered methylation in the promoter sequences of the respective genes. After confirming varied cell viability and significant changes in oxidative stress toward normal and high glucose exposure, followed by a significantly high expression of ET-1 gene in high glucose samples, we hypothesized reduced methylation in the promoter of ET-1 gene. To check our hypothesis we analyzed 175 CpGs at 25 CpG sites in HUVECS treated with normal and high glucose concentrations. Out of 75 CpGs, we found only 36 methylated CpGs in cell treated with normal glucose (20.5% methylation). To our understanding it was quite low methylation however we were expecting further reduction after cells being exposed to high glucose concentration. Surprisingly, the outcome was not as we expected. In cells treated with high glucose, showed almost similar number of methylated CpGs (30 methylated CpGs out of 175) which makes 17.1% methylation in comparison to 20.5% methylation in normal glucose treated HUVECs (Figure 6). Cells with low glucose concentrations exhibited high methylation at CpG positions 22, 251, 236 and 380. At positions 92, 155, 247, 295 and 314, no methylation was found. Cells treated with high glucose concentrations, showed high methylation at positions 22, 74 and 260. No methylation was detected at positions 34, 100, 251, 289 and 305.

24 and 48 h exposure of cells to glucose demonstrated a time dependent effect on cell viability and oxidative stress. ET-1 gene expression was also affected in a time dependent manner. However we used only maximum (48 h) exposure for the methylation analysis, as it has shown the maximum effect on cell viability and reactive oxygen species.

Our data conclude that cells treated with high and low glucose concentrations demonstrate no significant difference in the promoter methylation of ET-1 gene. In hyperglycemic conditions, the significant changes in the expression of ET-1 gene might be due to other factors. Oxidative stress and hyperglycemia may regulate the gene expression by recruiting other proteins.

## Conclusion

Our study demonstrate a significantly high expression of ET-1 gene in response to high glucose exposure in HUVECs. It also reports that hyperglycemic condition leads to elevated oxidative stress and reduce cell viability. Upon high glucose exposure, no significant changes were noticed in the promoter methylation of ET-1 gene.

## Data Availability

The original contributions presented in the study are included in the article/supplementary material, further inquiries can be directed to the corresponding authors.
